# Supplementary material for: Regional prevalence of hypertension among people diagnosed with diabetes in Africa, a systematic review and meta-analysis
Source: PLOS Glob Public Health. 2023 Dec 5;3(12):e0001931. doi: 10.1371/journal.pgph.0001931 (PMC10697518; doi:10.1371/journal.pgph.0001931)
Supplement: S1 Table — (PDF) [file pgph.0001931.s002.pdf]

Search string

Initial Search conducted in 2021

PUBMED

Hits	Terms	Results
#1	((((incidence) OR (prevalence)) OR (comorbidity)) OR (risk factors)) OR (treatment outcomes)	**5,234,789**
#2	(Africa) OR (African Countries)	**408,896**
#3	(hypertension) OR (high blood pressure)	**698,900**
#4	((Diabetes type 1) OR (diabetes type 2)) OR (diabetes mellitus)	**528,125**
#5	((((Africa) OR (African Countries)) AND (((((incidence) OR (prevalence)) OR (comorbidity)) OR (risk factors)) OR (treatment outcomes))) AND ((hypertension) OR (high blood pressure))) AND (((Diabetes type 1) OR (diabetes type 2)) OR (diabetes mellitus))	**1,932**

**HINARI**	Results
(\(Diabetes type 1\) OR \(diabetes type 2\) OR \(diabetes mellitus\)) AND (\(hypertension\) OR \(high blood pressure\)) AND (\(incidence\) OR \(prevalence\)\) OR \(comorbidity\)\) OR \(risk factors\)\) OR \(treatment outcomes\)) AND (\(Africa\) OR \(African Countries\))	**309**

EMBASE

#	Searches	Results
1	exp Diabetes Mellitus, Type 1/ or exp Diabetes Mellitus, Type 2/	4356786
2	type 1 diabetes.mp.	673454
3	type 2 diabetes.mp.	253271
4	T1D$.mp.	2746793
5	T2D$.mp.	567102
6	1 or 2 or 3 or 4 or 5	493900
7	exp Hypertension/	897111
8	hypertension.mp.	1074644
9	blood pressure.mp.	709508

10	7 or 8 or 9	1551520
11	incidence.mp.	1486883
12	prevalence.mp.	1347198
13	risk factor$.mp.	1634832
14	determinant$.mp.	353900
15	comorbidity.mp.	437491
16	11 or 12 or 13 or 14 or 15	2307386
17	exp Africa/	400563
18	africa$.mp.	238367
19	17 or 18	532078
20	6 and 10 and 16 and 19	1566

Updates search run on March 2023

Pubmed

Hits	Terms	Results
#1	((((incidence) OR (prevalence)) OR (comorbidity)) OR (risk factors)) OR (treatment outcomes)	**6,037,184**
#2	(Africa) OR (African Countries)	**459,336**
#3	(hypertension) OR (high blood pressure)	**730,175**
#4	((Diabetes type 1) OR (diabetes type 2)) OR (diabetes mellitus)	**588,325**
#5	((((Africa) OR (African Countries)) AND (((((incidence) OR (prevalence)) OR (comorbidity)) OR (risk factors)) OR (treatment outcomes))) AND ((hypertension) OR (high blood pressure))) AND (((Diabetes type 1) OR (diabetes type 2)) OR (diabetes mellitus))	**1,967**

**HINARI**	Results
(\(Diabetes type 1\) OR \(diabetes type 2\) OR \(diabetes mellitus\)) AND (\(hypertension\) OR \(high blood pressure\)) AND (\(incidence\) OR \(prevalence\)\) OR \(comorbidity\)\) OR \(risk factors\)\) OR \(treatment outcomes\)) AND (\(Africa\) OR \(African Countries\))	**332**

EMBASE

#	Searches	Results
1	exp Diabetes Mellitus, Type 1/ or exp Diabetes Mellitus, Type 2/	432210
2	type 1 diabetes.mp.	81872
3	type 2 diabetes.mp.	253271
4	T1D$.mp.	32486
5	T2D$.mp.	82472
6	1 or 2 or 3 or 4 or 5	493900
7	exp Hypertension/	897111
8	hypertension.mp.	1074644
9	blood pressure.mp.	709508
10	7 or 8 or 9	1551520
11	incidence.mp.	1486883

12	prevalence.mp.	1347198
13	risk factor$.mp.	1724904
14	determinant$.mp.	353900
15	comorbidity.mp.	437491
16	11 or 12 or 13 or 14 or 15	4429426
17	exp Africa/	402196
18	africa$.mp.	430147
19	17 or 18	644856
20	6 and 10 and 16 and 19	2066
