# Supplementary material for: Regional prevalence of hypertension among people diagnosed with diabetes in Africa, a systematic review and meta-analysis
Source: PLOS Glob Public Health. 2023 Dec 5;3(12):e0001931. doi: 10.1371/journal.pgph.0001931 (PMC10697518; doi:10.1371/journal.pgph.0001931)
Supplement: S2 Table — (PDF) [file pgph.0001931.s003.pdf]

Table 2: Quality Assessment of Studies included in the review

**Full citation**	**Are there clear research questions?**	**Do the collected data allow to address the research questions?**	**Is the sampling strategy relevant to address the research question?**	**Is the sample representative of the target population?**	**Are the measurements appropriate?**	**Is the statistical analysis appropriate to answer the research question?**	**Quality Score**
Abdelbagi, et al (2021)	Yes	Yes	Can't Tell	Yes	Yes	Yes	**4**
Berraho et al (2012)	Yes	Yes	Yes	No	Yes	Yes	4
Choukem et al. (2007)	Yes	Yes	Yes	No	Yes	Can't Tell	4
Dagnew et al (2019)	Yes	Yes	Yes	Yes	Yes	Can't Tell	4
Demoz et al (2019)	Yes	Yes	Yes	Yes	Can't Tell	Yes	4
Makwero et al (2018)	Yes	Yes	Yes	No	Yes	Yes	4
Mengesha (2007)	Yes	Yes	Yes	No	Yes	Can't Tell	3
Mogre et al (2014)	Yes	Yes	Yes	No	Yes	Yes	4
Regassa et al. (2020)	Yes	Yes	Yes	Yes	Yes	Can't Tell	4
Adeniyi et al. (2016)	Yes	Yes	Yes	No	Can't Tell	Yes	3
Arije et al. (2007)	Yes	Yes	Yes	Yes	No	Can't Tell	3
Githinji et al (2018)	Yes	Yes	Yes	Yes	Yes	Yes	5
Ndege et al. (2014)	Yes	Yes	Yes	No	Yes	Yes	4
Thinyane et al (2013)	Yes	Yes	Yes	No	Yes	Can't Tell	3

Abdissa (2020)	Yes	Yes	Yes	Yes	Yes	Yes	5
Akalu et al (2020)	Yes	Yes	Yes	Yes	Yes	Yes	5
Awadalla et al (2017)	Yes	Yes	Yes	Yes	Yes	Yes	5
Chetty et al (2021)	Yes	Yes	Yes	Yes	Yes	Yes	5
Gezawa et al. (2019)	Yes	Yes	Yes	Yes	Yes	Yes	5
Hussein et al (2020)	Yes	Yes	Yes	Yes	Yes	Yes	5
Kemche et al (2020)	Yes	Yes	Yes	No	Yes	Yes	4
Tsegaw et al (2021)	Yes	Yes	Yes	Yes	Yes	Yes	5
Unadike et al (2011)	Yes	Yes	Yes	Yes	Yes	No	4
Wanjohi et al (2002)	Yes	Yes	Yes	No	Yes	Yes	4
Amankwah-Poku et al (2020)	Yes	Yes	Yes	Yes	Yes	Yes	5
Danquah et al (2012)	Yes	Yes	Yes	Can’t Tell	Yes	Yes	4
Kalain & Omole 2020	Yes	Yes	Yes	Yes	Yes	Yes	5
Goie & Naidoo 2016	Yes	Yes	Yes	Yes	Yes	Can’t Tell	4
Thomas et al 2013,	Yes	Yes	Yes	Yes	Yes	Yes	5
Boake & Mash 2022	Yes	Yes	Yes	Can’t tell	Yes	Can’t Tell	3
Rotchford & Rotchford 2002	Yes	Yes	Yes	Yes	Yes	Yes	5

Mfeukeu-Kuate et al. 2022	Yes	Yes	Yes	NO	Yes	Yes	5
Amoussou-Guenou et al. 2015	Yes	Yes	Yes	Yes	Yes	Can’t Tell	4
Ovono et al 2011	Yes	Yes	Yes	Yes	Yes	Yes	5
Kimando et al 2017	Yes	Yes	Yes	Yes	Yes	Yes	5
Chahbi et al 2018	Yes	Yes	Yes	Can’t tell	Yes	Yes	4
Dzudie et al 2012	Yes	Yes	Yes	Yes	Can’t tell	Yes	4
Kilonzo 2017	Yes	Yes	Yes	Yes	Yes	Can’t Tell	4
Munyogwa et al. 2020	Yes	Yes	Yes	Yes	Yes	Yes	5
Mwita et al 2012	Yes	Yes	Yes	Can’t tell	Yes	Yes	4
Kahloun et al.2014	Yes	Yes	Yes	Yes	Yes	Yes	5
