# Supplementary figures and images for: Regional prevalence of hypertension among people diagnosed with diabetes in Africa, a systematic review and meta-analysis
Source: PLOS Glob Public Health. 2023 Dec 5;3(12):e0001931. doi: 10.1371/journal.pgph.0001931 (PMC10697518; doi:10.1371/journal.pgph.0001931)

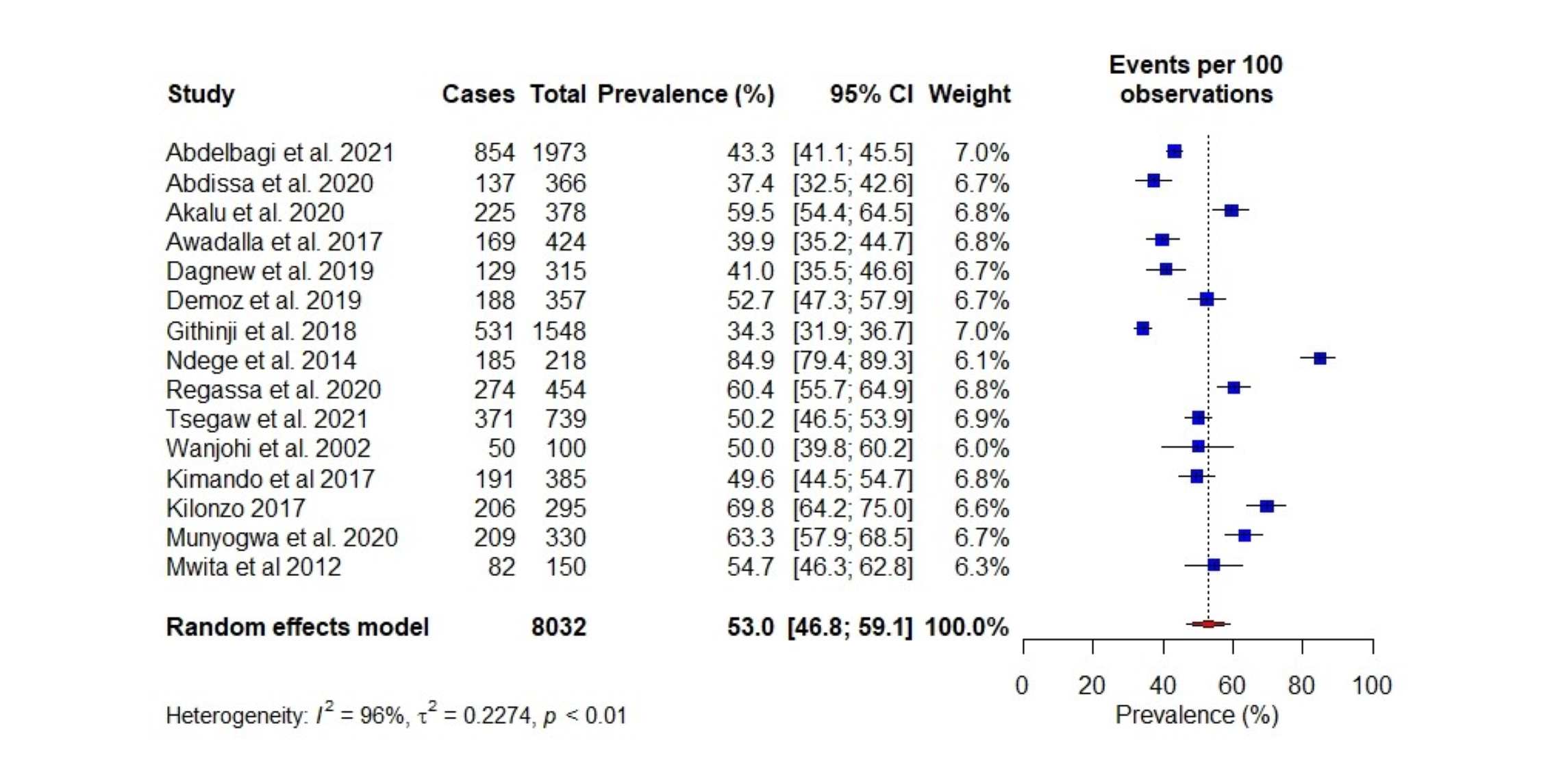

Supplement: S1 Fig — (TIF) [file pgph.0001931.s004.tif]

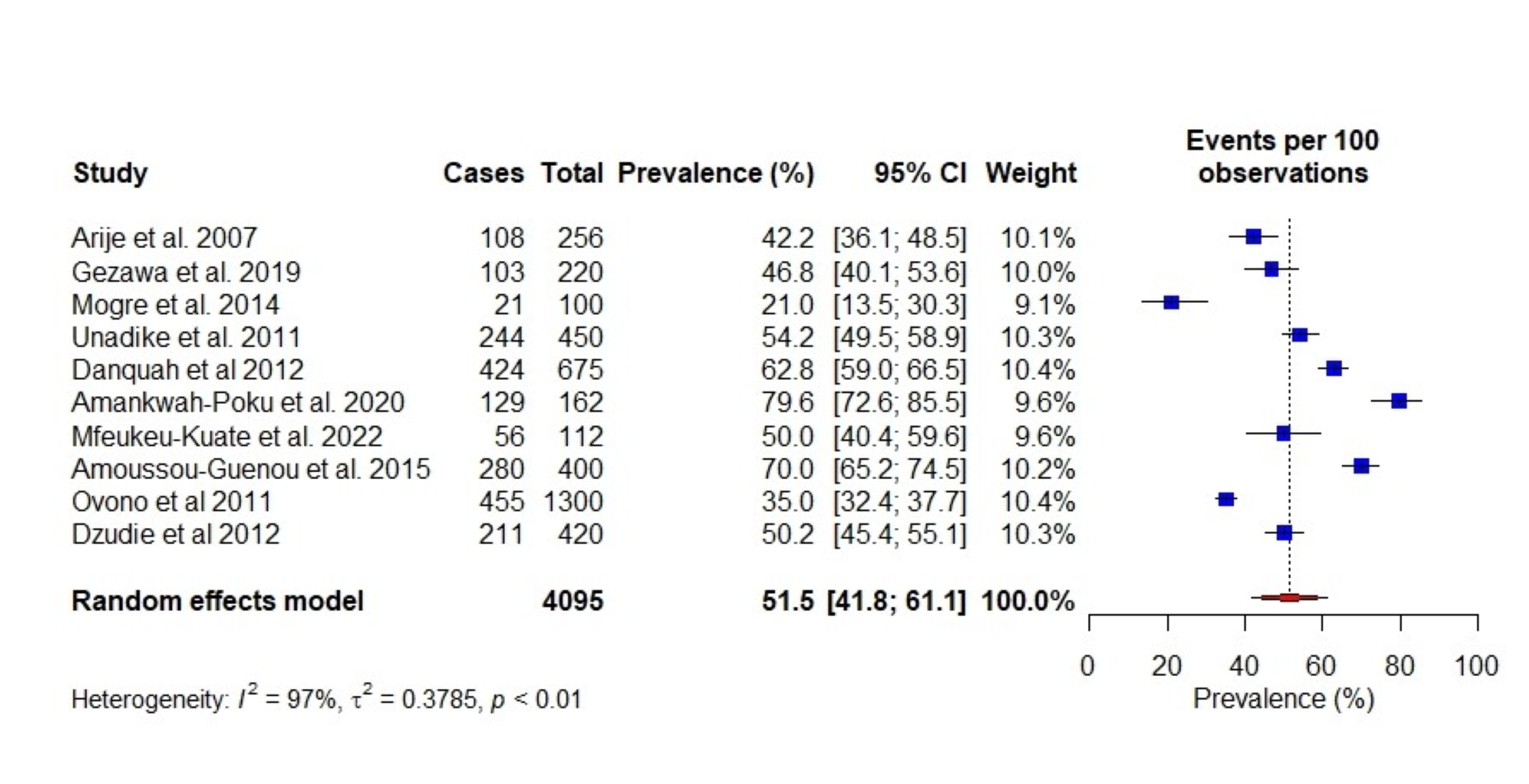

Supplement: S2 Fig — (TIF) [file pgph.0001931.s005.tif]

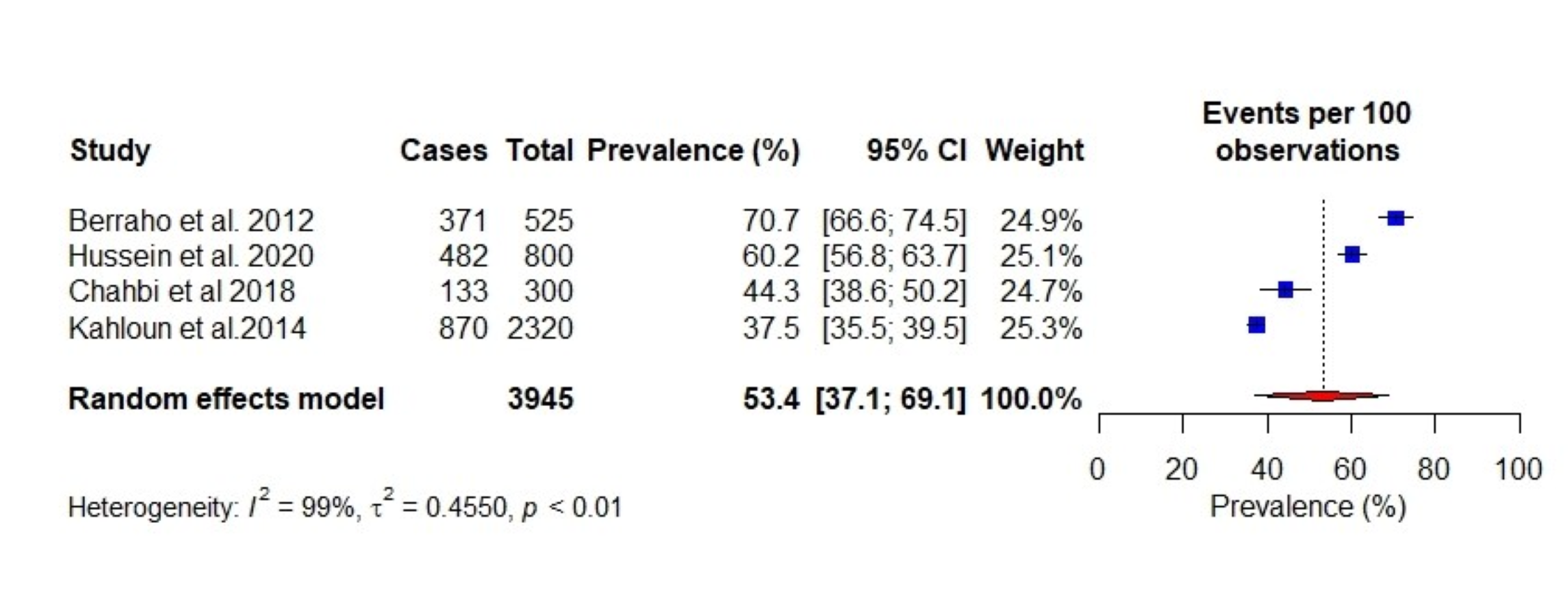

Supplement: S3 Fig — (TIF) [file pgph.0001931.s006.tif]

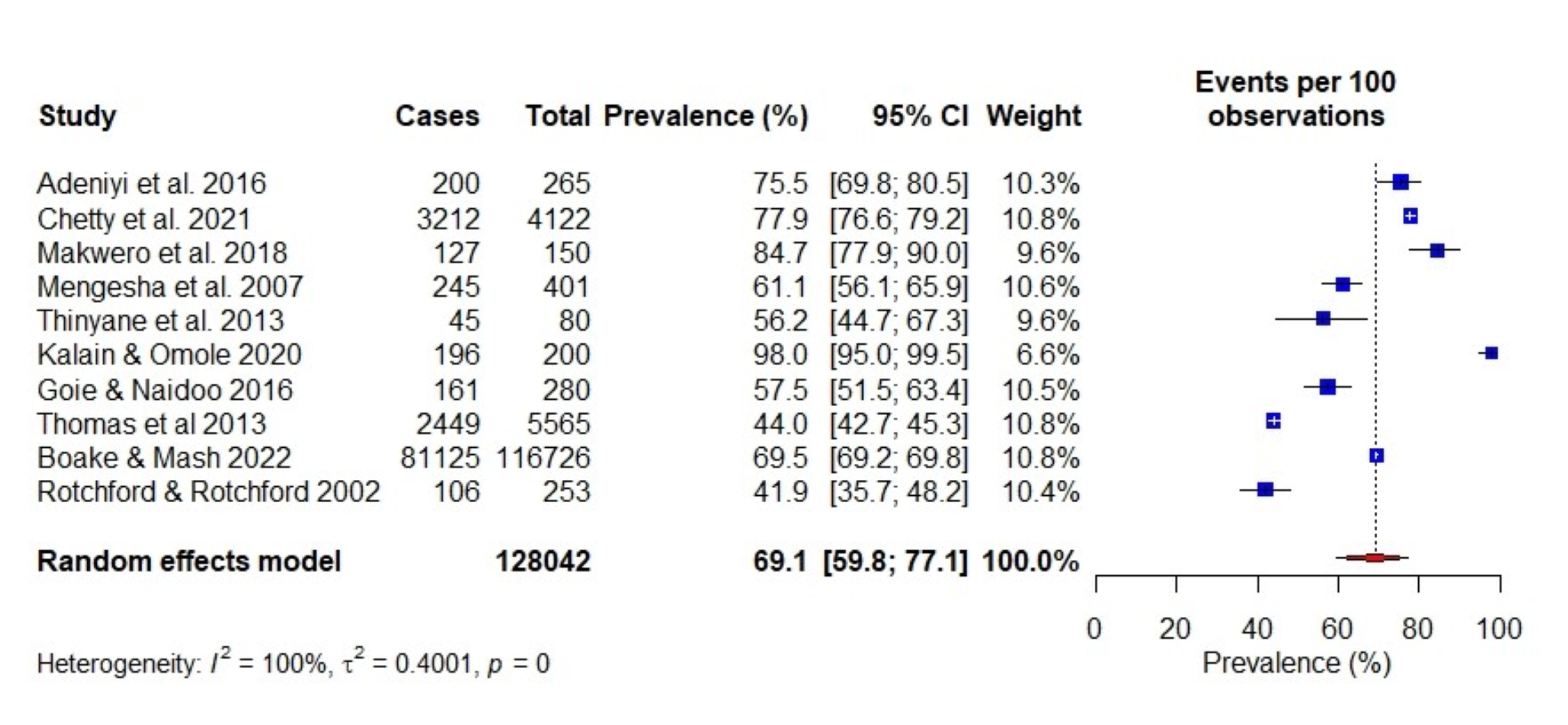

Supplement: S4 Fig — (TIF) [file pgph.0001931.s007.tif]

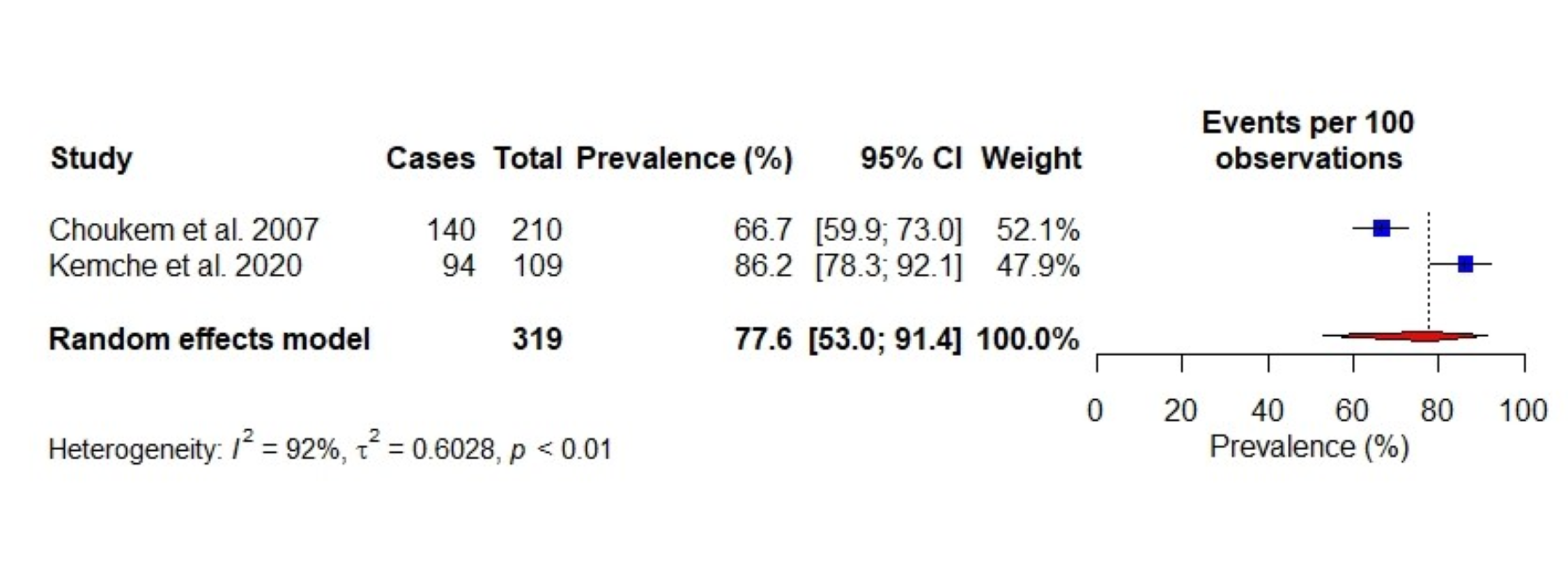

Supplement: S5 Fig — (TIF) [file pgph.0001931.s008.tif]

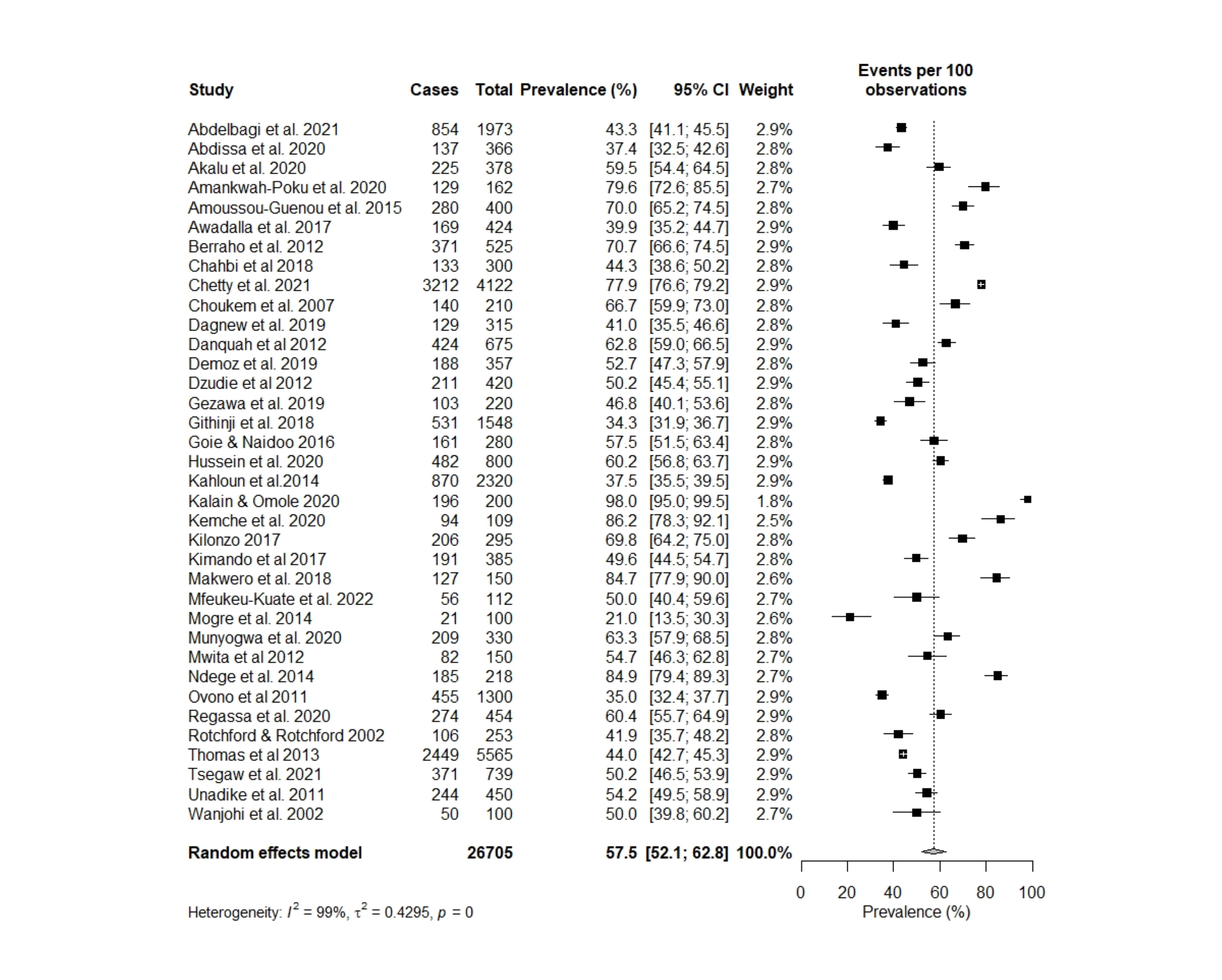

Supplement: S6 Fig — (TIF) [file pgph.0001931.s009.tif]

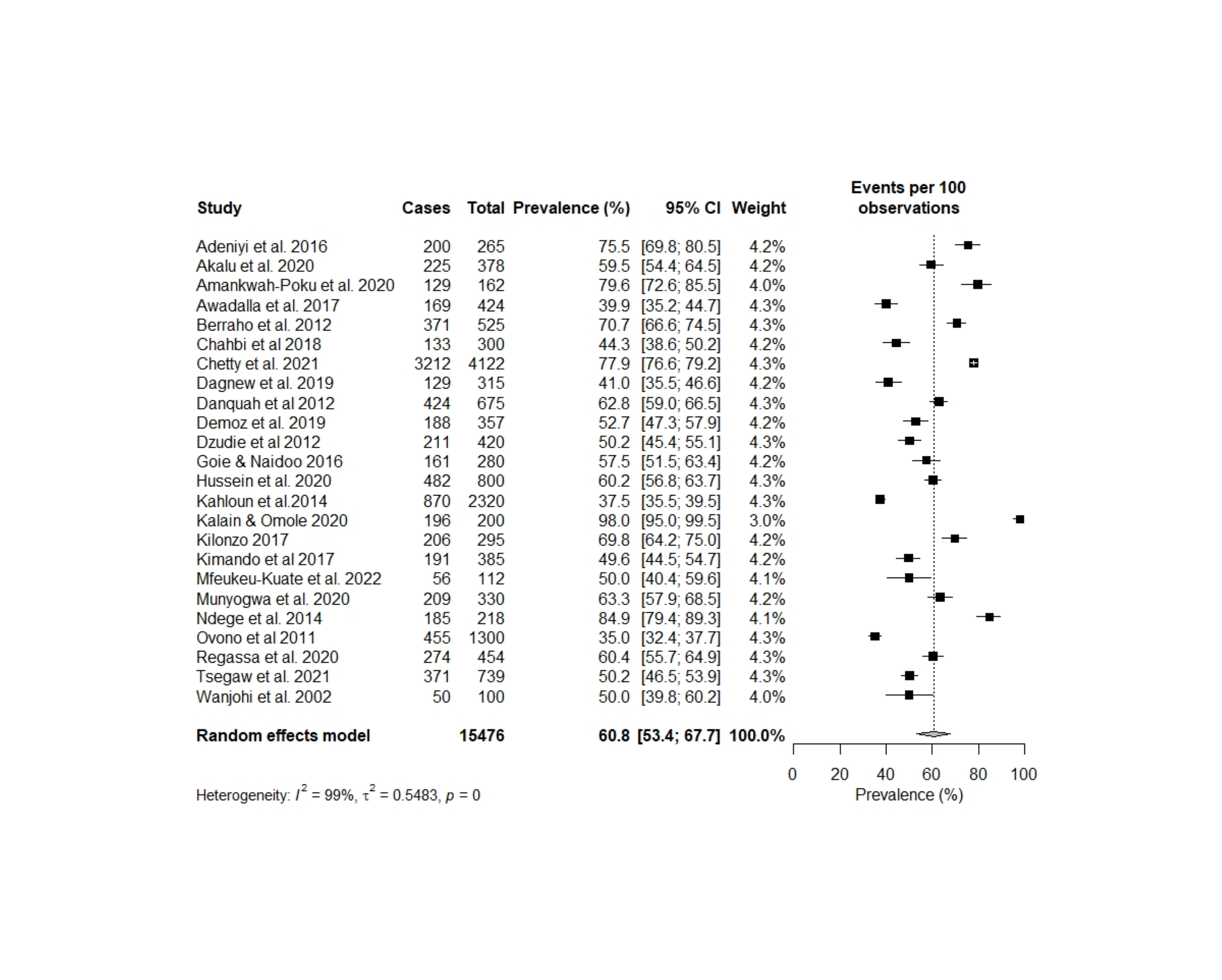

Supplement: S7 Fig — (TIF) [file pgph.0001931.s010.tif]
