# Supplementary material for: Regional prevalence of hypertension among people diagnosed with diabetes in Africa, a systematic review and meta-analysis
Source: PLOS Glob Public Health. 2023 Dec 5;3(12):e0001931. doi: 10.1371/journal.pgph.0001931 (PMC10697518; doi:10.1371/journal.pgph.0001931)
Supplement: Attachment — Submitted filename: Reviewer Response .docx [file pgph.0001931.s011.docx]

Reviewer #1

**1. The study rationale for estimating the prevalence of HTN among diabetes is neither clear from the introduction nor a necessary one. I believe it is more important to estimate the prevalence of joint occurrence of HTN and DM in the population. So instead of using Diabetics as the study population, I would suggest having the entire population as the study population and estimate the prevalence of co-occurrence of HTN and DM in the general population. The search strategy given by the authors may be sufficient for this objective.**

**Response.** Thank you for this comment. Firstly, we have revised the introduction to communicate this rationale clearly. This point was extensively discussed among the authors during the initial conception of this study. Our earlier searches yielded almost few to no papers that examined the co-prevalence of hypertension and diabetes in the general population. Again, that was not our objective. Our aim was to estimate the prevalence of hypertension among patients with diabetes to highlight morbidity in this population. We believe that our findings could highlight the need to implement interventions that would address the needs of patients who live with both hypertension and diabetes. We went into this study with the understanding that patients diagnosed with diabetes have a higher risk for other CVD complications if they have comorbid hypertension. Essentially, our focus was not on the general population but rather on this high-risk population.

**Question 2. For prevalence studies, the standard is to include representative surveys conducted in the community. It is not clear whether the authors included only such studies or whether they included hospital or facility-based studies as well.**

**Response**: Given the limited nature of the community-based studies in the region and our quest to comprehensively capture the burden of hypertension among populations with diabetes, we found only institutional-based studies assessing the prevalence of hypertension among people with diabetes.

This was highly anticipated a priori given that resources are generally limited in the African region to conduct a community-level study that assesses the prevalence of hypertension among people with diabetes. This may be practically uncommon in Africa.

This assertion is also validated by earlier systematic reviews that used institutional-level studies to assess the prevalence of hypertension among this population. However, we have highlighted that studies in this review are institution-based.

Please see below

[**Ethiopia**](https://www.ncbi.nlm.nih.gov/pmc/articles/PMC10153558/#:~:text=The%20prevalence%20of%20hypertension%20among%20type%202%20diabetes%20mellitus%20(DM,from%2032%25%20to%2082%25.) no community-based study

[**China**](https://academic.oup.com/inthealth/advance-article/doi/10.1093/inthealth/ihad047/7224139) (only three community-based studies were found).

**3. For LMICs, Google Scholar is an important database to search for published/unpublished literature. I suggest running a search in Google Scholar for additional studies.**

**Response:** Thank you for this comment. Based on the advice of the informationist, we included the HINARI database given that the platform is freely available for researchers in LMICs as part of the WHO effort to enhance health research in LMICs. This database contains several grey literature just like Google Scholar. Based on this assumption the study team decided to use HINARI to increase our chances of pulling in several eligible grey literature. Nonetheless, we have highlighted the limited number of databases as a limitation.

**Question 4. The eligibility criteria do not mention types of study designs that will be included in the review.**

**Response**: We used only institution-based cross-sectional studies and we have clarified this in eligibility criteria. In a number of systematic reviews that attempted to quantify the prevalence of chronic conditions such as CVDs, only cross-sectional study designs were used, Moreover, examining the prevalence of a phenomenon is an intrinsic property of cross-sectional design(See studies below)

<https://journals.plos.org/plosone/article?id=10.1371/journal.pone.0239929>

**Question 5. MMAT is not the correct tool for assessing the quality of cross-sectional surveys in SR, if that was the study design included in the review.**

**Response:** The mixed method Appraisal tool is suitable for different study designs. It has specific versions tailored for critically appraising different study designs. Given that this review focused on only cross-sectional designs, we applied the component of the tool that assesses the risk of bias for cross-sectional designs. This component focuses specifically on sample representativeness, sampling clarity, measurement, and analytic appropriateness of the studies which are key sources of bias for cross-sectional studies. Even though the name suggests as if it is for only mixed method studies, it is rather applicable for all study designs. Its validity and suitability for different study designs have been extensively explored and have been used by several studies.

See below

<https://www.sciencedirect.com/science/article/pii/S0895435618300829>

**Question 6. In statistical methods, the authors have mentioned GLM with logit transformation. It is not clear why this methodology was used. For a simple prevalence meta-analysis, it is not required to do a logit transformation.**

**Response**: Thank you for highlighting this. We have included and emphasized our use of the DerSimonian-Laird’s random-effects model to compute pooled prevalence.

**Question 7. There is no mention of the high level of heterogeneity in the pooled estimate.**

Response: We have highlighted the high prevalence in the results and discussions sections and have also stated it as a limitation. We have admitted in the result that there was high heterogeneity among studies.

**Minor comments**

**Question 1. Abstract is not written as per the PRISMA guideline. [https://doi.org/10.1371/journal.pmed.1001419]**

**Response:** Thank you for drawing our attention to this: We have revised the abstract to be consistent with the PRISMA Guide. We have also revised the abstract to meet the unstructured abstract style of the journal

**Question 2. Mean age cannot be calculated in this manner in a SRMA. A simple average of extracted mean ages from each study is not statistically correct.**

**Response:** we computed the mean and standard deviation which is often used by SRMA. We have however included the age range to provide further clarification of the age of the study participants. Additionally, we have also included the age range and average reported by the studies in Table 1.

**Question 3. It is not possible in this study to determine the risk factors for HTN or DM and how the p-values were calculated for this is not written.**

**Response:** We did not calculate p-values for the factors associated with hypertension among those with diabetes. Rather, the factors were narratively synthesized across relevant studies. We have made this clearer in the “synthesis” section of the manuscript.

**Question 4. In the introduction, the authors have used the word prevalence and proceed to numbers in millions. Prevalence has to be given as a proportion or percentage per unit of population or instead the word prevalence may be avoided.**

Response: Thank you for highlighting this. This has been addressed in the introduction. We have extensively revised the introduction to make it clearer.

**Question 5. Table 1 is not cited in the main text. Main tables should not be provided in the supplement but in the main text itself.**

Response: This has been addressed. Table 1 has been cited.

Reviewer #2:

**The limited use of databases, limited language, and limited grey literature search make this review not as comprehensive as it could be, and this should be noted in limitations**.

**Response #:** We have highlighted the limited number of databases as a limitation.

**Question: There are areas of repeated text in Lines 117-120 and • Lines 120-121 and Lines 124-126 are paraphrased but essentially the same.**

**Response:** We appreciate this comment. This has been addressed, and repetitive lines deleted.

**Question: I could find no reference in the body of the results to Table 1.**

Response: Thank you very much for drawing our attention to this. This has been resolved, Table 1 has been referenced**.**

**For clarification**

**Question 1. Kindly explain what the “population, outcome, region” strategy is. It is quoted as if a standard. Can you provide a reference for where it is derived or explain how the authors derived the strategy if it de novo.**

**Response**: Thank you for bringing this up. We derived this from the JBI guide for framing research questions. This has been clarified in the manuscript.

**Question 2. The authors state that the Hypertension cut-off was specified as 140/90 and 130/80. Can you reference the guidelines used to make this specification? Under what circumstances did you accept the various definitions? The latest guidelines of the 2022 ADA recommend that BP should be controlled to <140/90 mm Hg in patients with diabetes mellitus, but <130/80 mm Hg if there is a higher CV risk with existing ASCVD or 10-year ASCVD risk of ≥15%. I suspect that the authors used older guidelines and should provide reference and clarity on the circumstances (comorbidities) under which they accepted the various cut off points**.

**Response:** Thank you for highlighting this. We used the [WHO cut off point](https://www.who.int/news-room/fact-sheets/detail/hypertension#:~:text=Hypertension%20is%20diagnosed%20if%2C%20when,days%20is%20%E2%89%A590%20mmHg.)), which is the current practice guidelines for many LMICs and particularly in the region we studied. The papers reviewed were not US-based and based on the WHO-recommended guidelines for countries in this region, it was reasonable to use the context-specific cut points. Evidently, almost all studies in this review also used the same criteria. The guidelines used to make this specification has been referenced in the manuscript.

**Question 3. Line 165-166: Kindly indicate where it was applicable to use MESH terms? On which terms in PUBMED as this used? Was it used on some and not others since the authors say, “where applicable”?**

**Response:** Thank you for drawing our attention to this. Largely, PubMed uses Mesh terms, and this has been clarified.

**Question 4. The search strategy stating only “Africa” or African countries seems limiting to me. Was NESH used here?**

**Response:** Thank you for bringing this up. This was discussed extensively with an informationist, and we used Africa or African Countries with Mesh terms on PubMed. HINARI is traditionally a database for LMICs particularly Africa and so all studies pooled were largely from Africa or African Countries. Additionally, in our second search, we entered the individual countries on HINARI and EMBASE and found not much of a difference compared to when we used the Africa or African countries MESH terms.

**Question 5. Why was the MMAT used for risk of bias assessment? Given that the study focused heavily on prevalence which would be primarily quantitative studies can authors justify the use of this tool which is usually for mixed methods**?

**Response:** The mixed method Appraisal tool was developed to critically appraise several study designs (it is not specific to mixed methods designs). It has specific versions tailored for critically appraising different study designs. Given that this review focused on only cross-sectional designs, we applied the component of the tool that assesses the risk of bias for cross-sectional designs. This component focuses specifically on sample representativeness, non-response bias, measurement, and analytic appropriateness of the studies which are key sources of bias for cross-sectional studies. Even though the name suggests as if it is for only mixed method studies, it is rather applicable for all study designs. Its validity and suitability for different study designs have been extensively explored and has been used by several studies <https://www.sciencedirect.com/science/article/pii/S0895435618300829>

**Question. Line 222-223 states: “32 (78%) of the studies utilized a cut-off point of >140/90 mm Hg for hypertension diagnosis, some 3 (7.3%) used >130/80 mm Hg as the cut-off.” What did the other studies use as their cut-off?**

Response: Thank you very much. We have clarified this to include studies that did not specifically state the threshold or cut-off for hypertension diagnosis.

**Question 7. Can the authors explain what was done when the strategy was updated? I understand that the search was repeated but I am not clear on how this was matched against what already existed. Was the title and abstract screening repeated? Was there a duplicate comparison between the old and new list? Using what software?**

**Response:** We followed the same process used in the initial search. We conducted title and abstract screening independently as was done in the initial search. The final eligible studies were compared with the initially included studies. There were duplicates and were excluded accordingly. Five new studies were identified and added to the initial 36 studies collected. We used Rayyan Software for the two processes. This is demonstrated in search two in the Prisma flow chart.

**Major changes requested.**

**Question 1. Even though the authors mention in one sentence in the abstract the use of I2 to assess heterogeneity, no such assessment is described in the actual text of the study. Please describe how heterogeneity was assessed. How was I2 used? What were the cut-off values? Did you also conduct a qualitative assessment of heterogeneity? Please describe more fully the methods of the main text**.

Response: We have elaborated this under the synthesis section of the study. We used I^2^ at 10% statistical significance to test heterogeneity but did not set any threshold or cut off based on recommendation.

**Question 2: Please describe the results of the risk of bias assessment. This is a critical component of systematic reviews and should not be a footnote in the methods. How did the risk of bias assessment affect the use of the studies? Did the scores influence the authors decisions on which studies should be in the meta-analysis? For example, given the high heterogeneity, were attempts made to do a meta-analysis on studies with only the best quality assessment scores? I highly recommend that the authors conduct meta-analyses only on studies with risk of bias of 4/5 to determine the prevalence in these low-risk studies. This would be a suitable form of sensitivity analysis especially as the I2 scores are so high.**

**Response:** Thank you very much for this comment. We have provided a further description of the risk of bias assessment. Again, no study was excluded based on the risk of bias score. As suggested, we conducted further analysis for studies that had a risk of bias score of 4/5 and found a nearly similar prevalence rate which is 57.5% compared to the overall prevalence rate of 58.1%. This has been added as a **S4 fig and S5 Fig**.

<https://www.ncbi.nlm.nih.gov/pmc/articles/PMC6383865/>

**Question 3. Since age emerged qualitatively as a major factor in hypertension prevalence, consider also doing a meta-analysis in two major age groups, again it may help reduce heterogeneity and provide a clearer picture of prevalence that could inform health care access planning.**

Response: Thank you for this comment. Even though this would have been helpful, we believe the way the ages were reported were quite diverse across studies. Others used range as well as median and mean which will make any kind of categorization difficult.

**Question 4. I caution how the authors interpret their findings. They state that their paper shows evidence of ever-increasing prevalence of hypertension and given the lack of trending data, it does not show this. There was no previous systematic review in Africa for comparison, so I recommend perhaps simply stating that the burden is high as opposed to increasing.**

**Response: Thank you for this comment.**  Even though it is admissible that the prevalence of hypertension is relatively higher in Africa compared to developed countries (based on recent global hypertension reports), we agree the findings of our review may be limited in demonstrating trends among this high-risk group. We have taken steps to clarify this language.

**Question: Limitations/ The lack of grey literature search and limited to only three databases is a weakness of the study that should be acknowledged in the limitations section. Especially given that the authors found publication bias.**

**Response:** Thank you for this comment. Based on the advice of the informationist we included the HINARI database given that the platform is freely available for researchers in LMICs as part of WHO's effort to enhance health research in LMICs. This database contains several grey literature just like Google Scholar. Based on this assumption the study team decided to use HINARI to increase our chances of pulling in several eligible grey literature. Nonetheless, we have highlighted this limited number of databases as a limitation.
